# The Role of Personal Opinions and Experiences in Compliance with Mass Drug Administration for Lymphatic Filariasis Elimination in Kenya

**DOI:** 10.1371/journal.pone.0048395

**Published:** 2012-11-19

**Authors:** Doris W. Njomo, Mary Amuyunzu-Nyamongo, Japheth K. Magambo, Sammy M. Njenga

**Affiliations:** 1 Eastern and Southern Africa Centre of International Parasite Control (ESACIPAC), Kenya Medical Research Institute (KEMRI), Nairobi, Kenya; 2 African Institute for Health and Development, Nairobi, Kenya; 3 Institute of Tropical Medicine and Infectious Diseases, Jomo Kenyatta University of Agriculture and Technology, Nairobi, Kenya; Tulane University School of Public Health and Tropical Medicine, United States of America

## Abstract

**Background:**

The main strategy adopted for Lymphatic Filariasis (LF) elimination globally is annual mass drug administration (MDA) for 4 to 6 rounds. At least 65% of the population at risk should be treated in each round for LF elimination to occur. In Kenya, MDA using diethylcarbamazine citrate (DEC) and albendazole data shows declining compliance (proportion of eligible populations who receive and swallow the drugs) levels (85%–62.8%). The present study's aim was to determine the role of personal opinions and experiences in compliance with MDA.

**Methods/Findings:**

This was a retrospective cross-sectional study conducted between January and September 2009 in two districts based on December 2008 MDA round. In each district, one location with high and one with low compliance was selected. Through systematic sampling, nine villages were selected and interviewer-based questionnaires administered to 965 household heads or adult representatives also systematically sampled. The qualitative data were generated from opinion leaders, LF patients with clinical signs and community drug distributors (CDDs) all purposively selected and interviewed. Sixteen focus group discussions (FGDs) were also conducted with single-sex adult and youth male and female groups. Chi square test was used to assess the statistical significance of differences in compliance with treatment based on the records reviewed.

The house-to-house method of drug distribution influenced compliance. Over one-quarter (27%) in low compared to 15% in high compliance villages disliked this method. Problems related to size, number and taste of the drugs were more common in low (16.4%) than in high (14.4%) compliance villages. Reasons for failure to take the drugs were associated with compliance (p<0.001). The reasons given included: feeling that the drugs were not necessary, CDD not visiting to issue the drugs, being absent and thinking that the drugs were meant for only the patients with LF clinical signs. A dislike for modern medicine prevailed more in low (6.7%) than in high (1.2%) compliance villages. Experience of side effects influenced compliance (*P*<0.001). The common side effects experienced included giddiness, fever, headache and vomiting. Social support, alcohol and substance use were not associated with compliance in both types of villages (p>0.05).

**Conclusions/Significance:**

Community sensitization on treatment, drugs used, their regimen and distribution method involving all leaders should be strengthened by the Programme Implementers. The communities need to be made aware of the potential side effects of the drugs and that health personnel are on standby for the management of side effects in order to build confidence and increase the compliance levels.

## Introduction

Lymphatic filariasis (LF) caused by filarial worms and transmitted by mosquitoes is ranked as the second largest cause of disability in the world [1]. Infection leads to a variety of clinical manifestations, including lymphoedema of the limbs and the genitalia (especially hydroceles) and elephantiasis and about 41 million people worldwide have visible symptoms [2]. A further 76 million have hidden infections, most often with microfilariae in their blood and hidden internal damage to their lymphatic and renal systems and about 44 million infected patients have recurrent infections and abnormalities of renal functions [3].

Lymphatic filariasis was identified as a potentially eradicable disease by the International Task Force for Disease Eradication in 1993[4].For elimination to occur, at least 65% treatment coverage of endemic communities and sustaining such coverage for 6–10 years is recommended [5]. In addition, no group of persons should remain totally untreated as groups that remain untreated form a reservoir of microfilaraemia (mf) contributing to transmission of the infection [6]. The elimination campaign is faced with the challenge of persuading people who have no symptoms of the disease to take the drugs [3] and to continue complying for as long as necessary.

In Kenya, MDA by community-directed treatment (ComDT) approach was launched in Kilifi district in 2002 and extended to Kwale and Malindi districts in 2003. During the subsequent rounds i.e. in 2005 and 2008, the ComDT approach was not applied due the financial constraints that were being experienced by the programme. The District Medical staff and politicians were first to be sensitized about MDA (endemicity of the area, purpose of mass treatment, drugs used, method of distribution, length of distribution and role of WHO in the programme) followed by peripheral health staff and community leaders. The community leaders then sensitized the community members and together they selected the CDDs based on an agreed upon criteria; they must be able to read and write; keep records; be trustworthy; be well known by the village members and be willing to distribute drugs to all eligible persons in the allocated areas without remuneration by the project. Using house-to-house method, the CDDs within a single day were expected to distribute the annual single-dose treatment of albendazole and DEC to all eligible members, and keep records. The dosage of DEC was based on age where 2 to 5 year old children were meant to take one tablet, those of 6 to 9 years, 2 tablets, 10–14 years, 3tablets and persons of over 14 years, 4 tablets. For albendazole, the dose was one tablet (400 mg) for all persons above 2 years of age. During the distribution exercise, the health personnel were on standby to manage side effect cases which are usually minor and include nausea, headache, dizziness, fever, malaise, decreased appetite and vomiting. The sensitization carried out during the 2005 and 2008 rounds was not as aggressive as that conducted in 2002 and 2003 when the ComDT approach was used. The community members only participated in selecting the CDDs and not in any other decisions regarding the implementation of the Programme as expected in the ComDT approach.

The Programme data for three MDA rounds in Kwale and Malindi districts show declining compliance levels,(proportion of eligible populations who receive and swallow the drugs)from 85% to 71% to 64.3% and 77% to 76% to 62.8% respectively. The present study's aim was to determine the role of personal opinions and experiences in compliance with MDA. The psychological definition of opinions, which are subjective beliefs and the results of emotion or interpretation of facts which may be supported by arguments by Damer [7] was used. Experience as a general concept refers to knowledge of something gained through exposure to that thing [8].

The study was conducted between January and September 2009 and a review of the existing literature on compliance factors in mass drug administration programmes was used to inform the design and analysis of the survey. The compliance factors therefore investigated were; provider-related, drug-related and client-related. The provider-related factors were mainly to do with the drug delivery method and included interaction with CDDs, time spent waiting for the CDD, delay in receiving supplies and in the process of MDA, and community sensitization and mobilization. The drug-related factors included the size, number and taste of the drugs, and experience of side effects related to the use of the drugs. The client-related factors were mainly to do with reasons for not taking the drugs in the 2008 MDA round and willingness to take the drugs in subsequent rounds and included perceived need for the treatment and perception towards modern medicine. Social support and alcohol and drug use and abuse were other client-related factors investigated.

## Methods

### Ethics statement

Ethical clearance was received from the Kenya Medical Research Institute (KEMRI)/National Ethical Review Board (Protocol Number 1077) and written informed consent sought from all the study participants. All the participants were adults above the age of 18 years and therefore no parents/guardians were expected to give consent on behalf of a minor.

### Study area

Kwale District, 40 km south of Mombasa, the second largest city in Kenya, has an area of 8360 km2 with a population of 649,931 persons [9] and lies at an altitude of between 60 and 135 meters above sea level. Malindi District is located 120 kilometers northeast of Mombasa, and lies between latitudes 2.2° and 4° south and between longitudes 39° and 41° east. It covers a geographical area of 7,605 km2 with a total population of 384,643 [9]. Both districts are endemic of LF caused by *Wuchereria bancrofti* and studies conducted in villages of Kwale district have reported 16.0% and 16.4% microfilaria prevalence [10]; [11]. The villages along the River Sabaki in Malindi have a filarial endemicity of at least 7.1% [12]. Kwale district has 3 hospitals, 5 health centers, 37 government dispensaries, and 3 private dispensaries. Accessibility of health services is however low. Majority of the population live over 5 kms to the nearest health facility. Shortage of drugs andlack of diagnostic facilities like X-Ray machines adversely affect provision of quality health care. Cost of health care system is also a barrier to access to services. The doctor/patient ratio stands at 1∶82,690 which in itself is telling of services offered due to shortage of staff in the health facilities. The utilization of health facility for child delivery stands at 32% [13], the main reasons for low levels of use being distances to the nearest health facilities and low socio-economic status. Malindi District has 3 hospitals (1 government and 2 private); 24 dispensaries (17 government and 7 NGO) and 4 private chemists. The average distance to the nearest health facility for urban areas is 1 km and 3 kms for rural areas. Most of the health facilities are therefore not accessible to the majority of the population. High poverty levels, cost sharing and long distances inhibit people from visiting these facilities. The doctor/patient ratio is 1∶19,502. The most prevalent diseases are; malaria, respiratory diseases, diarrhea, intestinal worms, STIs, anemia and eye infections. The utilization of health facility for child delivery is at 41% and reasons for low usage are distance and low socio-economic status [14].

### Study design and setting

This was a retrospective cross-sectional study which utilized both quantitative and qualitative methods. Based on the December 2008 MDA Programme data, Tsimba location in Kwale district was selected for high (80% and above) and Gadini for low (below 60%) compliance and in Malindi District, Goshi location represented high and Gongoni low compliance. Systematic sampling was applied to select four villages (Kavunyalalo, Magongoloni, Midodoni and Zhogato) to represent Malindi District and five villages (Patanani, Mbengani, Tzunza, Takawa and Dzivani) to represent Kwale District.

### Study population

A total of 965 households were then selected through systematic sampling and interviewer-based questionnaires administered to household heads or adult representatives by trained research assistants for quantitative data. For qualitative data, in-depth interviews were conducted with 80 LF patients with clinical manifestations, 80 opinion leaders and 15 CDDs all purposively selected. The selection of the patients was based on their having clinical signs of either hydrocele and/or lymphoedema while that of the opinion leaders was based on their being leaders of social, political and religious groupings. With regard to the CDDs, their selection was based on their having distributed the drugs during the 2008 MDA round. To elicit more information on opinions of MDA, sixteen focus group discussions (FGDs) were carried out with adult and youth male and female single-sex groups and moderated by the lead author assisted by trained field assistants using *Kigiriama* and *Kiduruma*, the local languages. Notes were taken during the FGDs and audiocassettes used to tape record all the information in the local languages. The tapes were later transcribed and translated into English.

### Statistical analysis

The quantitative data were analyzed using SPSS version 16.The responses to open-ended questions were coded before entry. Equivalent responses were pooled to arrange the responses in different categories. Two-way tables were used to compare categorical data and the statistical significance of differences in compliance was assessed by the χ^2^test.A *P* value of ≤0.05 was considered statistically significant. The quantitative data was collected before the qualitative data. This was mainly to generate meaning for the various patterns observed from the preliminary quantitative data analysis. The qualitative data from various sources were analyzed manually according to the themes of the study and triangulated for cross verification. The triangulation helped increase the credibility and validity of the results by continuously cross-checking the data from the various sources. The data were examined separately for clusters that recorded high and low compliance. Similar questions were asked to various types of respondents and data were triangulated in order to check for consistency and divergence of views. The study's dependent variable was compliance with treatment and the independent variables were personal opinions and experiences.

### Background characteristics of the study participants

The mean age of the household heads or representatives most (62.6%) of whom were female, was 39.5 (SD = 15.6) and median, 35.0 (range 18–92 years). A large majority (45.8%) had never attended school, while30.7% attended but did not complete primary school level. Over three-fifths (62.5%) were peasant farmers and one-fifth (24.7%) casual laborers, fishermen, business owners or salaried workers. Slightly more than one-fifth (12.7%) of the respondents were housewives (Table 1).

**Table 1:** Distribution of household heads by background characteristics.

Background Characteristic	
Age (n = 965)	Frequency (%)
15–24	150 (15.5)
25–34	293 (30.3)
35–44	203 (21.0)
45–54	141 (14.6)
> = 55	178 (18.5)

Education (n = 965)	Frequency (%)
Never attended school	442 (45.8)
Incomplete primary	308 (30.7)
Completed primary	172 (17.8)
Completed secondary	22 (2.3)
Tertiary education	22 (2.3)

Main Occupation (n = 965)	Frequency (%)
Peasant farmer	603 (62.5)
Housewife	123 (12.7)
Casual laborer	88 (9.1)
Small/big business	74 (7.6)
Fisherman	44 (4.6)
Salaried	33 (3.4)

About one-quarter (24.6%) of the opinion leaders interviewed were local leaders, 23.2%, Christian religious leaders and another 23.2%, social group leaders. Islamic leaders and traditional herbalists accounted for 5.8% and 4.3% respectively. Teachers and policemen represented 18.9% of this group.

Nearly two-thirds (64%) of the LF patients had hydrocele, 35%, lymphoedema and only 1% had both manifestations. The mean age of the patients was 52.4 years; (SD = 16.7) the youngest was 22 and the oldest 98 years old. Slightly more than two-thirds (67.5%) were male, one-half was from high and one-half from low compliance areas.

The FGDs participants included single sex adult (35 years and above) and youth (18 to 34 years) male and female respondents of homogenous characteristics. Each FGD contained a minimum of 8 and a maximum of 12 participants and standard procedure [15] were adhered to.

## Results

### Opinion on drug distribution method

0The respondents' opinion towards the house-to-house method of drug distribution influenced compliance. A higher proportion (27%) in low compared to 14.9% in high compliance villages disliked this method of drug distribution, *P*<0.001. Reasons given for dislike of the method were significantly associated with compliance, *P*<0.001. Most of the reasons were around the CDDs. The CDDs' failure to explain the need for the drugs and their side effects were the leading reasons given for the dislike of the distribution method. Other reasons included having to wait for the CDD for a long time and poor CDD interaction (Table 2).Furthermore, in 4 FGDs from high and 4 from low compliance villages, a large majority of the participants reported that the interaction with the CDDs was poor as they did not give adequate information about the drugs, left drugs behind for absentees, did not have good communication skills, ‘overdosed’ the people and were strangers to the community members. One female adult FGD participant in a low compliance village stated that:

*One thing which surprised me this last time is that the CDDs came to my house when I was not in but gave my children the drugs, the worst thing is that my neighbour's children were in my house then and were also given the drugs, then I was blamed. Next time CDDs must ensure that they issue drugs in the presence of an adult and if there is no adult they must come to check again the following day.*

(42 year old, female adult respondent, Zhogato village)

**Table 2:** Reasons for not wanting the drugs to be distributed in the same way.

Reasons Given	Low Compliance	High Compliance
	N (%)	N (%)
Had to wait too long for the CDD	33 (6.1)	4 (1.0)
CDD did not explain need for drugs and side effects	51 (9.5)	15 (3.7)
CDD did not have enough drugs	2 (0.4)	1 (0.2)
Poor interaction with the CDD	27 (5.0)	20 (5.0)
CDD never came	13 (2.4)	19 (4.7)
Have another illness	1 (0.2)	0 (0)
Never heard of MDA	1 (0.2)	0 (0)
No Complaint	409 (76.2)	344 (85.4)
Total	537 (100)	403 (100)

Low compliance: less than 60%.

High compliance: 80% and above.

The study results however showed that both groups of opinion leaders and of patients liked the present process of drug distribution but emphasized the need to involve all stakeholders and to do proper planning. A 45 year old village elder in a high compliance village (Patanani) stated that:

*I accept the house-to-house method of drug distribution because in my record of the community members almost everybody took the drugs but all leaders should be involved in such projects as it makes bringing people together easier.*

A 98 year old LF patient in one of the low compliance villages further stated that:

*According to me, the house-to-house method of drug distribution is good because I did not have to walk long distances for the drugs. I was relaxing at home for it to be brought by the CDDs.*

A 37 year old primary school teacher in Takawa, a low compliance village furthermore highlighted on the importance of educating the communities by stating that:

*The Ministry of Health in collaboration with the NGOs should educate experts who will be educating people in their villages about the importance of mass drug administration.*

### Opinion on drugs size, number and taste

Problems related to size, number and taste of the drugs were significantly associated with compliance, *P*<0.01. A high proportion (16.4%) in the low compared to 14.4% in high compliance villages reported to have had problems related to the size, number and taste of the drugs. Majority of the FGD participants in 4 high and 4 low compliance villages reported that one type of drug was too big to be swallowed especially by children. Moreover, some discussants in 4 FGDs in low compliance villages felt that the number of drugs administered were too many while some members of 9 FGDs (6 in low and 3 in high compliance villages) reported that the drugs were too bitter and emitted a bad smell which elicited nausea. A female respondent in one FGD in a low compliance site stated that:

*The drugs made many people feel like vomiting, they were too many and another person instead of taking all four drugs at once, he kept them and took one each day for he said that they were too many (and the group laughed).*(20 year old, female youth respondent, Tsunza village)

### Experience of side effects

Experience of side effects was associated with compliance, *P*<0.001. Three-fifths (60%) in the low compared to 41% in the high compliance villages reported having experienced side effects. There was also a significant association between the type of side effects and compliance with treatment *P*<0.001. The side effects which included giddiness, fever, headache and vomiting were more expressed in the low than in the high compliance villages. Furthermore, some participants of 6 FGDs, (4 in low and 2 in high compliance villages) reported that their community members experienced problems of increased swelling of genitals and blisters. Moreover 3 CDDs in low and one in a high compliance village indicated that some community members complained of swollen limbs and testicles, painful male private parts and sexual inactivity/low libido. Slightly more than one-tenth (12.8%) of the 51 patients with hydrocele moreover reported that the swelling of their genitals came as a result of taking the drugs. A 50 year old male LF patient in Patanani, a high compliance village stated that:

*One week after taking the drugs, I started feeling pain in the whole body and afterwards I found my genitals swelling and shrinking from time to time and it continued up to date. It has now developed into a hydrocele. Thus I believe that the drugs made me develop a hydrocele instead.*

### Reasons for not taking treatment during the 2008 MDA

**Figure 1:**
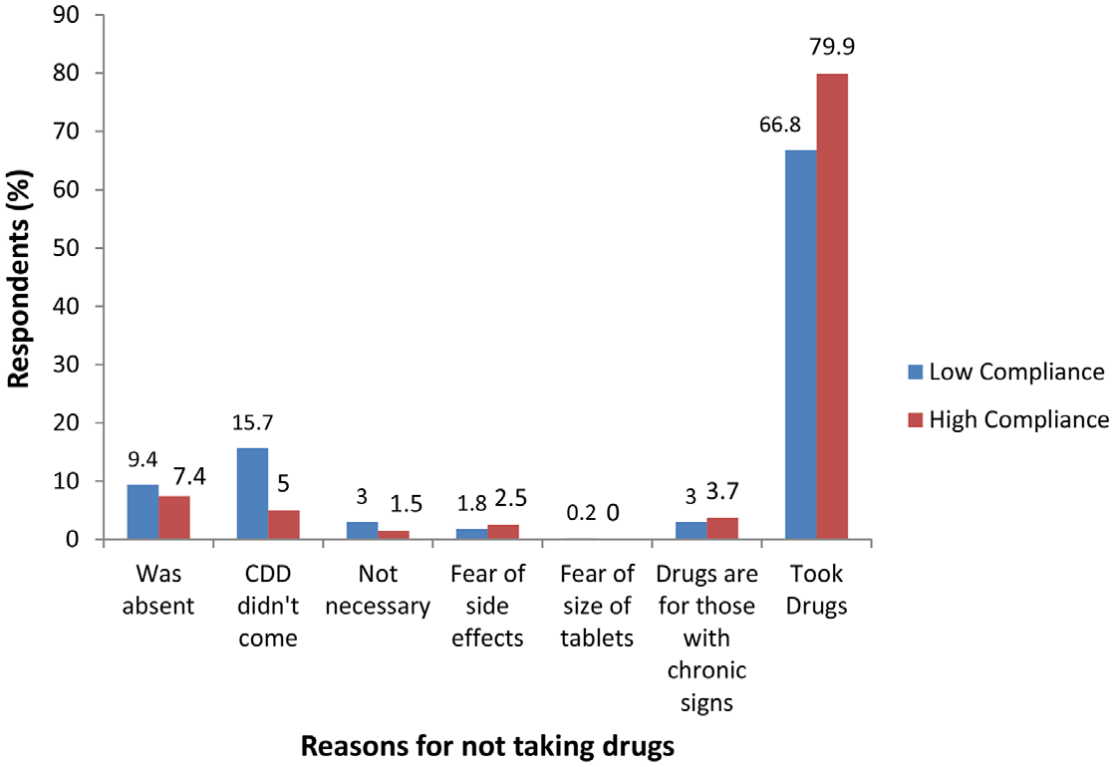
Reasons for not taking drugs during MDA. Low compliance: less than 60%. High compliance: 80% and above.

The reasons for failure to take treatment during 2008 MDA were associated with compliance, *P*<0.01. Reasons such as the CDD not visiting to administer the drugs and feeling that the drugs were not necessary prevailed more in low than in the high compliance villages (15.7% compared to 5% and 3% compared to 1.5% respectively). Other reasons for not taking drugs during 2008 MDA included being absent and thinking that the drugs were meant for only the patients with LF clinical signs (Figure 1).

### Willingness to comply in subsequent MDA rounds

Willingness to take drugs during subsequent MDA rounds was significantly associated with compliance, *P*<0.001. Although both high and low compliance groups were fairly satisfied with MDAs, more (93.3%) household heads in high compared to 88.3% in low compliance villages were willing to take drugs in the subsequent MDAs rounds. Reasons why one would not take drugs in subsequent MDA rounds were significantly associated with compliance, *P*<0.01. The reasons reported include poor perception towards modern medicine and a lack of seeing the necessity of taking the treatment (Table 3).Most participants in the FGDs expressed their satisfaction with the MDAs but emphasized the importance of the role of information and education to compliance. One adult male participant in the high compliance site commented:

*Yes the village members are ready to take drugs but attaining knowledge and education about MDA before distributing the drugs will be very good.*(39 year old, male adult respondent, Mbengani village)

**Table 3:** Reasons given for unwillingness to take drugs in subsequent MDA.

Reasons Given	Low Compliance	High Compliance
	N (%)	N (%)
Not distributed in my house/village	19 (3.5)	1 (0.2)
Do not like modern medicine	4 (0.7)	1 (0.2)
Not necessary for me	11 (2.0)	4 (1.0)
Fear of reactions	6 (1.1)	7 (1.7)
Have another illness	2 (0.4)	3 (0.7)
Will take drugs	501 (92.3)	387 (96.0)
Total	543(100)	403(100)

Low compliance: less than 60%.

High compliance: 80% and above.

Furthermore, two-thirds (66.7%) of the patients in the low compared to 44.1% of those in high compliance villages indicated that the community member's participation was poor, due to inadequate social mobilization. A 70 year old female LF patient in one high compliant village (Mbengani) stated that:

*The Programme is good. What I would say next time the (MOH) should insist the chief and the village elders to pass information to the community members in good time so that they be alert.*

In Dzivani, a low compliant village in Kwale district, a 60 year old LFfemale patient indicated that:

*The drug distribution should be improved through educating people on the benefits of MDA and the need to use LF drugs.*

### Social support and alcohol and substance use

There was no significant association between compliance with treatment and social support, *P*>0.05. Close to three-fifths, 56.8% of the respondents in high and 56.6% in low compliance villages reported that they did not get any social support during MDA. However, a higher proportion (52.8%) of opinion leaders from the high compared to those from the low (47.2%) compliance villages indicated having facilitated MDA through awareness creation. Furthermore, a higher proportion (54.8%) of the patients in the low compared to 45.2% in the high compliance villages indicated that they facilitated the MDA through community mobilization.

Compliance with treatment was also not associated with alcohol and substance use. More than two-thirds (26.1%) of the respondents from high and 28.8% from low compliance villages reported that they use alcohol and substances.

## Discussion

The results reported in this paper indicate that the method of drug distribution has an effect on compliance. Majority of those with a dislike for the current drug distribution method were from the low compliance areas and claimed to have spent a lot of time waiting for the CDD or the distributor did not have sufficient quantities of drugs to administer. Babu and Kar [16] also found that delay in supplies as well as processes to undertake MDA in some places influenced the coverage and compliance. Another predominant reason for a dislike of the drug distribution method highlighted in the current study was poor interaction with the CDDs. In the qualitative research arm of the current study, Njomo *et al.*, [17] reported that the CDDs themselves viewed their number and length of the distribution period as inadequate for them to effectively conduct the exercise and to interact well with the community members. A study done in Cameroon by Wanji *et al.*, [18] related high compliance with doxycycline for onchocerciasis and loiasis with the fact that the drug was delivered by selected community members which guaranteed some trust and was a great motivating factor in the population and compliance with treatment. Selection of CDDs by the community was found to be a useful indicator for predicting sustainability and monitoring progress towards self-sustenance of drug delivery in Uganda [19].

The current study results also show that problems related to the size of the drugs, their number and taste as well as drug swallowing were more common in the low compliance communities. Similar reasons for non-compliance have been given in other programmes [20]; [16], which included the drugs being too many to be consumed at once.

The results of this study have demonstrated that although both low and high compliance groups considered the treatment to be necessary, a lack of perceived need for treatment was a more common reason for failure to take the drugs among the low compliance group. The results are similar to those reported by Babu and Kar [16], where lack of perceived benefits for the treatment and risk perception were found to be persistent in the community and contributed substantially to low compliance. Furthermore as indicated by Weerasooriya *et al.*, [21], other than adverse reactions other reasons attributed to failure to consume the drugs were the use of other medicines, a lack of feeling of the necessity for them and forgetting.

According to the results of the current study, a dislike for modern medicine prevailed more in the low compliance villages while reasons such as having another illness were more predominant among the high compliance villages. This finding concurs with the study of Lakwo and Gasarasi [22] which associated local beliefs and poor perceptions of modern medicine with non-adherence to treatment. Mathieu *et al.*, [23] also found that having been sick was one of the common reasons given for non-compliance.

Experience of side effects following consumption of the drugs contributed to low compliance. The study by Babu and Kar [16] also attributed fear of side-effects as the most important reason for not taking drugs after receiving them. Lack of information on side effects has been observed to be detrimental to the LF elimination programme. Similar studies by Ramaiah *et al.*, MacLaughlin *et al.*, Babu and Satyanarayana [24]; [25]; [26] have reported the effect of adverse side effects on coverage and compliance with MDA. As highlighted by Evans *et al.*, [27], health education in LF MDA is critical due the recurring issue of treatment with DEC which causes severe reactions in people with microfilaraemia (mf), triggered in some way by the death of mf and possibly an effect on the adult worms and since most people with mf have no clinical symptoms, these reactions are particularly unwelcome.

In Burkina Faso, Kyelem *et al.*, [28] further emphasized the need for Behaviour Change Communication (BCC) messages to address potential side effects and management guidelines for major symptoms following MDAs for the health personnel. These would help the health personnel to reduce related fears among the communities and perhaps raise the levels of compliance. Findings of the results of a study by Njomo *et al*., [29] showed that all the CDDs felt that there was inadequacy in source, content and frequency of informing the communities about MDA and called for a combined effort by health workers, local administration and mass media for improved compliance levels. In Zanzibar, where the programme has been successful, the communities were educated on importance of adverse reactions as evidence of the therapeutic action of the drugs and as a result the public was willing to accept the treatment [30]. Furthermore, Amarillo *et al.*, [31] highlighted the importance of informing people that they could be infected although the symptoms of the disease would appear five to ten years after infection which motivated the community members to submit to MDA even though they were asymptomatic and had not been diagnosed through nocturnal blood examination.

Social support includes real or perceived resources provided by others that enable a person to feel cared for, valued, and part of a network of communication and mutual obligation [32]. Social support can include perceived emotional support, instrumental support (e.g. direct assistance such as transportation), and informational support (e.g., sharing knowledge). In the current study, social support mainly focusing on being informed and encouraged by others in the community to take the drugs during MDA did not play a role in compliance. Similarly, Amarillo *et al.*, [31] did not associate being able to discuss LF and MDA with other people and being encouraged by others to take the drugs with compliance. However, Yirga *et al.*, [33] on factors associated with compliance with CDTI for onchocerciasis control in southern western Ethiopia associated social support with compliance.

Use and/or abuse of alcohol and other drugs carries risks as it impairs one's capacity to make proper judgment increasing their likelihood of missing treatment for LF. However, in the current study this behavior was not associated with non-compliance. Lakwo and Gasarasi [22] however associated alcohol use among community members with non-adherence.

Finally based on the discussion of the current study and that of other studies, for improved compliance there is a need to educate and mobilize the community members on all aspects of the Programme well in advance to the distribution time. All leaders and stakeholders need to be involved in making the community members understand the importance of taking the drugs and reassuring them that the expected side effects are minor and any severe cases should be reported to the health personnel. The drug distributors need to be adequately trained and their numbers increased for improved interaction and compliance.

## Conclusions

This study suggests specific measures to the Programme Implementers for increased compliance and successful MDA campaigns. First, there is a need for community involvement in the whole MDA process and the numbers of CDDs as well as the length of distribution period need to be increased for improved interaction with the community members. Secondly, more health education messages should affirm that repeated treatment prevents lymphatic filariasis and that signs of the disease may occur many years after infection. Thirdly, the importance of community sensitization on, drugs used, their regimen and related potential side effects should be emphasized. Lastly and very important is the need to involve all leaders in community mobilization for better awareness creation about MDA and its benefits.
